# Multi-Modal Ensemble Deep Learning in Head and Neck Cancer HPV Sub-Typing

**DOI:** 10.3390/bioengineering11010013

**Published:** 2023-12-23

**Authors:** Manob Jyoti Saikia, Shiba Kuanar, Dwarikanath Mahapatra, Shahriar Faghani

**Affiliations:** 1 Electrical Engineering, University of North Florida, Jacksonville, FL 32224, USA; 2 Department of Radiology, Mayo Clinic, Rochester, MN 55905, USA; kuanar.shiba@mayo.edu (S.K.); faghani.shahriar@mayo.edu (S.F.); 3 Inception Institute of Artificial Intelligence, Abu Dhabi 127788, United Arab Emirates; dwarikanath.mahapatra@inceptioniai.org

**Keywords:** ensemble deep learning, multi-modal CT/PET, head and neck cancer, squamous cell carcinoma, CNN

## Abstract

Oropharyngeal Squamous Cell Carcinoma (OPSCC) is one of the common forms of heterogeneity in head and neck cancer. Infection with human papillomavirus (HPV) has been identified as a major risk factor for OPSCC. Therefore, differentiating the HPV-positive and negative cases in OPSCC patients is an essential diagnostic factor influencing future treatment decisions. In this study, we investigated the accuracy of a deep learning-based method for image interpretation and automatically detected the HPV status of OPSCC in routinely acquired Computed Tomography (CT) and Positron Emission Tomography (PET) images. We introduce a 3D CNN-based multi-modal feature fusion architecture for HPV status prediction in primary tumor lesions. The architecture is composed of an ensemble of CNN networks and merges image features in a softmax classification layer. The pipeline separately learns the intensity, contrast variation, shape, texture heterogeneity, and metabolic assessment from CT and PET tumor volume regions and fuses those multi-modal features for final HPV status classification. The precision, recall, and AUC scores of the proposed method are computed, and the results are compared with other existing models. The experimental results demonstrate that the multi-modal ensemble model with soft voting outperformed single-modality PET/CT, with an AUC of 0.76 and $F_{1}$ score of 0.746 on publicly available TCGA and MAASTRO datasets. In the MAASTRO dataset, our model achieved an AUC score of 0.74 over primary tumor volumes of interest (VOIs). In the future, more extensive cohort validation may suffice for better diagnostic accuracy and provide preliminary assessment before the biopsy.

## 1. Introduction

Head and neck cancer is the sixth most common cancer worldwide and the eighth most common cancer in men [1]. Reported cases of cancers arising from the oropharynx have been increasing yearly. The majority of head and neck squamous cell carcinoma (HNSCC) arise primarily in the pharynx, oral cavity, sinonasal tract, larynx, and hypopharynx regions and remain a significant public health concern. The non-keratinizing stratified mucosal epithelium lining the upper aerodigestive tract is the origin of oropharyngeal squamous cell carcinoma (OPSCC). Over the past three decades, human papillomavirus (HPV)-related OPSCC has increased dramatically in developed countries and currently ranks as the most common cancer caused by high-risk HPV in the United States and Germany [2,3]. The important carcinogenic pathways leading to OPSCC are smoking, excessive alcohol consumption, and human papillomavirus (HPV) infection. HPV is considered one of the primary risk factors for squamous cell carcinomas, where 90% of HPV-positive oropharynx cancers are infected, with a high risk of type 16. On the other hand, HPV-negative cancers are often associated with patients of a little older age in the US population. Evidence indicates that compared to the HPV-negative form, HPV-associated OPSCC has distinct biological, anatomical, molecular, and clinical features, such as longer overall survival, improved treatment response, and a more favorable outcome [4,5]. Therefore, determining human papillomavirus (HPV) status for oropharyngeal cancer can be an essential diagnostic factor and an important factor for treatment decisions with new staging guidelines [6]. The diagnosis of HPV-related OPSCC is made by performing molecular testing on tissue specimens such as immunohistochemical (IHC) staining of p16, RNA in situ hybridization (ISH), DNA polymerase chain reaction (PCR) [7], or RNA reverse transcription PCR. These histology-based methods are invasive, time-consuming, expensive, and not broadly available. On the other hand, standard chemotherapy and radiotherapy treatment for early-stage cases may lead to diverse outcomes depending on several factors, such as overall tumor stage and location [8].

Image analysis for precision medicine has been widely used for better cancer diagnosis and treatment decisions and has made impressive advances in recent healthcare research. The radiographic scanning technique includes CT, MRI, and PET imaging to identify tissue structure patterns and localize suspicious lesions. On the other hand, histology imaging has been the gold standard for finding diagnostic insights and provides better molecular tissue features. The long-term goal of this diagnostic imaging and anatomic pathology imaging is to improve the treatment quality of individuals based on patient data. Therefore, an advanced prediction model characterizing disease heterogeneity will allow clinicians to make early treatment decisions in cancer care and help characterize phenotypes for various cancers. Recent studies have demonstrated the feasibility of quantitative texture feature analysis for predicting HPV status in OPSCC using CT scans [9]. Fluorodeoxyglucose (18F) PET and non-contrast CT scans are routinely used for treatment planning, staging, diagnosis, and surveillance of HNSCC carcinomas and provide imaging data amenable to quantitative data mining. Consequently, the intra-tumor heterogeneity quantification can be assessed by merging the anatomical or structural tissue density information from CT volumes and tumor metabolic activity provided by FDG-PET volumes for subsequent outcome predictions. Besides that, non-contrast CT scans provide standardized tissue density values for better texture heterogeneity evaluation.

Over the past decade, radiomics has emerged as a potential alternative for characterizing different tumor phenotypes and predicting cancer prognosis [10]. The growing number of studies indicates its possible capability for treatment analysis and extracting robust imaging features in personalized medicine [11,12]. In radiomics, texture, intensity, transform-based, radial, shape, size, and morphology features are extracted from heterogeneous intra-tumor regions and analyzed using machine learning models. The histogram and grey label-based matrix features are extracted from CT and PET images and quantify the spatial distribution of tissue intensities. The Wavelet and Laplacian of Gaussian filters are often applied during the feature extraction step [11]. Subsequently, the combination of CT and PET scans has shown superior visual interpretation in HPV detection and provides better diagnosis than CT or PET alone [13]. However, the radiomics approach has some limitations, including a large number of human-engineered and manual feature selections, lack of standardization across different modalities, feature reduction, and often the selection of algorithms. Therefore, accurate quantification of tumor heterogeneity has the potential to identify aggressive treatment plans for high-risk patients.

In recent years, advanced deep learning (DL) models have come forth for various computer vision tasks in medical image analysis. These learning-based methods can automatically extract low- and high-level features from the raw dataset with faster inference time [14], detect hidden motifs, and find inter-voxel relationships in a translation-invariant fashion. The 3D convolutional neural network (CNN)-based approaches can generally be classified into two categories: multi-frame-based methods from a single modality or the multi-modal approach with different fusion techniques. The multi-frame-based techniques exploit temporal correlations between adjacent frames in the image volumes and are relatively challenging since the inter-frame information cannot be evaluated directly. On the other hand, the multi-modal models are data-hungry and greatly depend on the large dataset size. Consequently, transfer learning-based techniques were introduced to use the pre-trained model, aggregate the image-level details by comprehensively leveraging the relationship among different modalities, and transfer such relationship knowledge to enhance the robustness of the proposed framework [15]. However, the availability of robust 3D models in OPSCC detection is scarce, with most models using 2D-based analysis or being pre-trained on natural RGB images. This paper aims to develop an effective 3D-based DL framework for OPSCC malignancy prediction from multiple image modalities.

Contributions: Segmenting small tumor regions and thereafter classifying them into different HPV sub-types presents a challenging problem in the field of oropharyngeal cancer. We acknowledge HPV detection as a machine-learning design problem for characterizing the local features and disease severity understanding. Therefore, a systematic analysis is introduced in our pipeline work, and the important contributions of our paper are summarized below.

- To identify the intra-tumor heterogeneity, an ensemble of 3D CNN models was targeted for our multi-modal feature learning from individual PET/CT volume and fused those features for final disease classification. Our weighted gate fusion technique follows a late fusion technique to extract dense feature maps from multiple sequences.
- Our training follows the multiple instance learning technique and creates multiple patches from tumor regions. Therefore, one can treat each subject as a bag of patches and each patch as an instance of the tumor zone [16].
- During training, each patch is assigned a label based on threshold techniques that consider how much overlap persists between the tumor region and the patch region. During testing time, instance-level prediction was performed across all patches, and majority and soft voting operations were performed for subject-level HPV status prediction.
- Our 3D model training procedure includes repeated cross-validation across five folds with stratification, evaluating sampling variability with standardization, and novel data pre-processing to control ensemble model bias.

## 2. Materials and Methods

### 2.1. Related Work

This section briefly discusses the prior deep learning work related to our proposed HPV detection task. In recent years, many deep learning-based approaches have made considerable advances and produced impressive achievements in CT/PET image analysis tasks like classification, detection, and segmentation. Bizzego et al. [17] demonstrated a unified radiomics and deep learning feature analysis on 3D/2D CT and PET tumor images through a RADLER classification pipeline and predicted the loco-regional recurrence in head and neck squamous cell carcinoma (HNSCC) with a better sensitivity and specificity outcome. Diamant et al. [15] introduced the seven-layered CNN model to predict the cancer outcome of patients with HNSCC on pre-treatment CT images. The framework validated the medical gray-scale images and has shown to complement their performance in several qualitative and quantitative ways, like AUC on distant metastasis (DM), AUC on loco-regional failure (LRF), and AUC on overall survival (OS). Le et al. [18] introduced a pseudo-volumetric CNN with a pre-processor module and self-attention model for predicting loco-regional recurrence, distant metastasis, and overall survival occurrence within a ten-year follow-up time frame for patients with squamous cell carcinoma. The model validated its performance on public and internal datasets and achieved an accuracy of 80% with an AUC of 0.69 across all outcomes. Naser et al. [19] proposed a series of DenseNet deep learning models, utilized 2021 HECKTOR Challenge PET/CT images and clinical data as separate input channels to predict the progression-free survival, and yielded a C-index value of 0.694, placing second in the competition. Lv et al. [12] introduced a multi-level fusion strategy that combined the image- and feature-level tumor information from CT/PET frames. They collected the head and neck cancer multi-center TCIA archive data and performed recurrence-free, metastasis-free, and overall survival analysis.

Data fusion from multi-modal sources and applying those into deep learning models has been successful in medical applications. Similar trends have been observed in recent medical imaging literature where different fusion paradigms leverage pixel, EMR, and EHR data for solving complex tasks that a single modality cannot readily tackle [20]. RGB and depth data fusion is essential in many tasks, such as refining object boundaries in object detection and indoor semantic segmentation. Cheng et al. [21] designed a late fusion layer to learn the weights over each modality in different scenes and merge features for object recognition. Li et al. [22] proposed a lightweight Dimensional Decomposition Residual network to address the 3D semantic RGB scene completion and dense prediction. They combined the depth and color image features in a multi-modal parallel fashion. They claimed that their novel factorized convolution and Atrous Spatial Pyramid Pooling module could aggregate information in multiple sequences with reduced network parameters.

### 2.2. Data Collection

**Figure 1:**
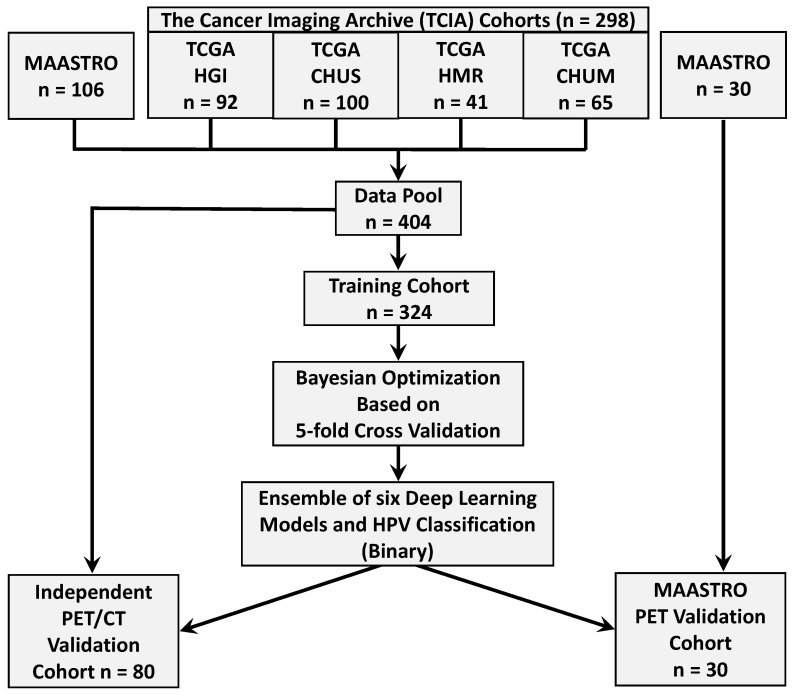
Data allocation summary and strategy of The Cancer Imaging Archive (TCIA), and MAASTRO data into model training, independent validation, and external validation cohorts.

**Figure 2:**
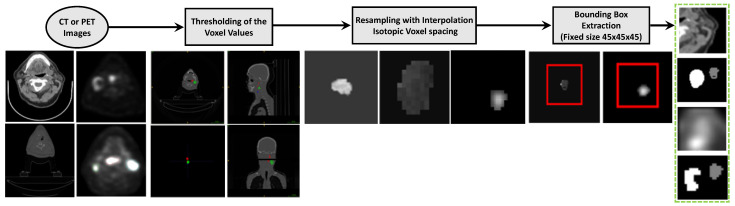
Pre-processing pipeline for input CT and PET NIfTI Images. The steps include thresholding, re-sampling the images to make them isotropic, windowing operation, and bounding box creation.

FDG-PET/CT images have been widely used in clinical practice for radiotherapy treatment planning. We collected 298 PET/CT imaging patient data from four different institutions in Québec [23] from (1) the Cancer Imaging Archive (TCIA) Public Access [24] including (a) 92 head and neck squamous cell carcinoma (HNSCC) patients treated at Hôpital général juif (HGJ) de Montréal, QC, Canada, (b) 100 patients treated at Centre hospitalier universitaire de Sherbrooke (CHUS), QC, Canada, (c) 41 patients treated at Hôpital Maisonneuve-Rosemont (HMR) de Montréal, QC, Canada, (d) 65 patients treated at Centre hospitalier de l’Université de Montréal (CHUM), QC, Canada, and (2) gathered 136 subjects of the “Head-Neck-Radiomics-HN1” collection from the Netherlands (“MAASTRO”) cohort [25,26]. The Cancer Imaging Archive (TCIA) collections host de-identified medical images and metadata information, and the providing institutions are responsible for consent and approvals. Each cohort has its own image acquisition settings and equipment, which is the cause of heterogeneity in image feature characteristics. Our data collection process includes imaging and metadata (Table 1) of 404 patients with primary squamous cell carcinoma tumors. Two subjects in the TCIA dataset were discarded because of post-processing segmentation issues. The data allocation steps are summarized in Figure 1. The OPSCC dataset provides PET and CT Dicom (NIfTI) files for each patient, and tumor volume segmentation masks were later created. The prepossessing pipeline is shown in Figure 2. For both non-contrast CT and PET images, the preprocessing pipeline includes intensity normalization, thresholding on the pixel values, resampling with interpolation to make isotropic voxel spacing (1 mm^3^), extracting 3D volume using the tight bounding box, and finally, obtaining the standardized image input for model training. It is observed that the slice thickness depends on the anatomy and structure being imaged [27]. The higher slice thickness spacing may introduce blurriness and decrease the spatial image resolution, subsequently impacting the 3D model’s performance. To mitigate their effects, we judiciously selected the PET/CT slice thickness as 1 mm^3^. We performed windowing operations on CT images for brightness and contrast enhancement and mapped the grey scale to restrict the HU threshold within a window level of 50 and a window width of 200. We re-sampled the voxels to uniform sizes to overcome the image data heterogeneity originating from different scanners and the rotational invariance of the texture features.

### 2.3. Tumor Segmentation and Registration

**Figure 3:**
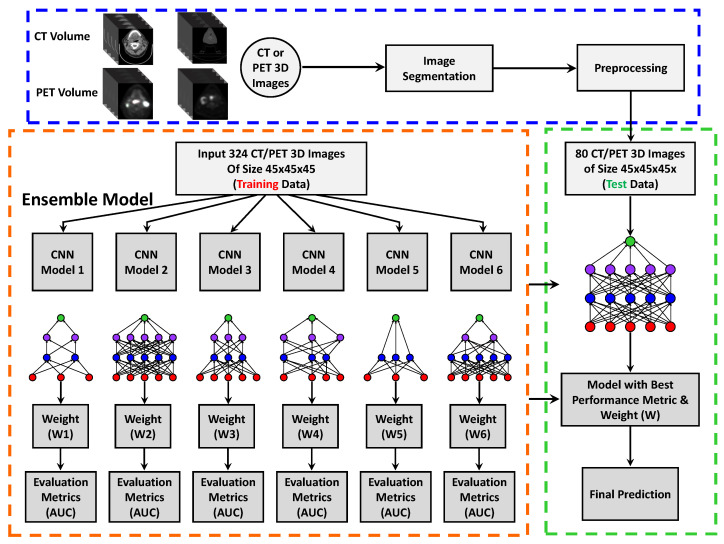
Pre-processing, image segmentation, and ensemble of 3D deep learning models. Ensemble learning combines the predictions from six CNN-based models and includes 5-fold cross-validation for error generalization.

Figure 3 summarizes the data segmentation, feature extraction, and classification workflow. In the current clinical application, physicians delineate tumor targets in CT images based on PET images and include the gross tumor volume. For our feature extraction, we separately defined CT and PET volume of interest (VOIs) in the primary tumor regions and segmented the volume of size 45×45×45 . Tumors outside the VOIs were ignored. Each tumor region was manually contoured on the PET/CT axial plane using the ITK-SNAP 3.8 segmentation module. The segmented masks and VOI files were stored in respective subject folders. Subsequently, the segmented images were verified, and label information was retrieved from the radiology metadata report. The ground truth (GT) manual segmentation agreement among raters was evaluated on fifteen subjects, and the dice coefficient values were noted between 0.8 and 0.85. Similarly, the agreement between the rater and the AI model was reported between 0.85 and 0.9. These high values show that the GT segmentation performed among raters was consistent with the deep learning model prediction and reliable for model evaluation.

The head and neck PET-CT image acquisition was performed separately from a single examination in the current scanning practice. Besides that, the image acquisition process of an organ takes a particular specific duration. Therefore, the diagnostic body part cannot be stationary during the image reconstruction step. To accurately align the corresponding tumor regions among multimodal images, it is necessary to register the image volumes before feature extraction. On the other hand, the tumor volumes defined on CT and those depicted on PET are not necessarily aligned and sometimes convey complementary information [28]. The streaking artifacts often form in CT images during the accusation process, resulting in high attenuation coefficients in corresponding PET regions. Sometimes, these high photon absorptions may lead to an overestimation of PET activity and produce high false positive findings. To overcome these temporal deformations across anatomical regions, the PET/CT images were segmented, and motion or streak artifacts were removed and co-registered across modalities. Before executing intensity-based registration, we excluded the uninvolved fat, bone, and air regions from the images. To accurately align the structures and overcome local errors, our non-rigid registration module used the simleITK-based 3D elastic transformation. The algorithm applied normalized mutual information (NMI) for the voxel similarity measure and modified Hausdorff distance (M-HD) as the performance metric.

## 3. CNN Architecture

**Figure 4:**
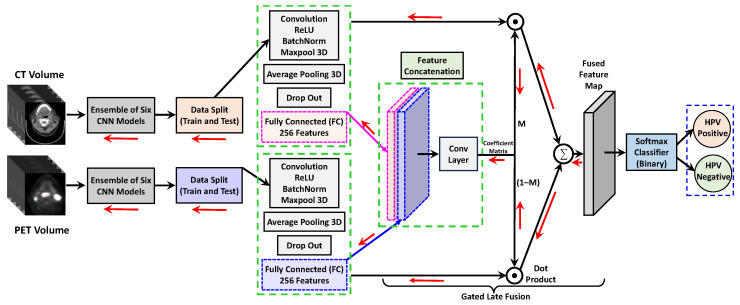
The proposed architecture diagram of our dual ensemble network for classification. Multi-modal feature concatenation combining CT/PET 3D volumes, and prediction of HPV binary classification in oropharyngeal squamous cell carcinoma. The ensemble inputs CT/PET volumes with patches of size 16×16×16 . The total number of fully connected layer features was concatenated from two channels to output a feature vector of size 512. The red arrow shows the back-propagation paths.

In recent years, the field of deep learning has advanced in various medical imaging tasks with the capability to extract features automatically from multi-modal and multi-scale architectures and increase performance in evaluations. Furthermore, our ensemble network uses a dual network and operates on different modalities. In this work, we have developed a 3D deep learning ensemble framework combining features from PET and CT imaging datasets. We have also performed HPV classification on the oropharyngeal cancer dataset. Figure 3 and Figure 4 shows the steps of pre-processing, training, testing, and prediction of HPV classification. Our ensemble architecture incorporates a series of five convolutional layers, ReLU, Batch Normalization, along with a linear combination of max and average pooling, drop out, and fully connected modules before feature concatenation in softmax classification. The proposed model uses different hyperparameters for training and optimizes the network parameters using the cross-entropy loss function and leaky ReLU optimizer. To overcome the vanishing gradient problem, a leaky ReLU non-linear activation function was applied to the non-positive filter output responses. The convolutional filters acted as feature extractors and adaptively learned the non-linear relationships in the input image kernel regions. We set out the different number of intermediate feature maps by repeatedly applying filters to intermediate layers. We finally down-sampled the input image features into smaller abstractions through max and average pooling operations.

To reduce computational complexity and improve weight sharing during back-propagation, a receptive field of 3×3×3 kernel was selected for convolution filter operations. The features extracted from the first three layers of the CNN were relatively noisy. Therefore, we introduced 2×2×2 max-pooling layers after each convolution operation and reduced the feature dimensions. The odd-sized filter (3) was selected to symmetrically divide the previous layer features around the current output features. Consequently, we followed two immediate convolutions and average pooling to improve the noise and edge blurring features and to handle lesion size variability and shifts in positions [29]. Our binary cross-entropy loss function learns to minimize the error in the probability distribution predicted by the model on the given dataset and the probability distribution in the training dataset. The dropout layer has been intruded on for regularization and minimizes the over-fitting problem during model training. Our proposed 3D deep learning ensemble framework combined six CNN models with different nodes in multiple layers to reduce model prediction variance. Using sampling and cross-validation methods, the model was trained on multi-institutional datasets. The layer-wise node variation was performed using different dropout and learning rate values and created multiple instances of the same CNN model. Finally, the feature concatenation layer assembled the discriminative patch representations and fused feature maps to another intermediate vector and performed binary classification using the soft-max classifier.

As described in Table 2, we trained Inception V3 [30], ResNet–50 [31], and DenseNet [32] 2D models for our result comparison. The models were loaded with pre-trained ImageNet weights, and then the last few layer weights were fine-tuned during training through transfer learning. The InceptionV3 model incorporates a deeper architecture with several stacked 1×1 convolutions and allows efficient computation through dimensionality reduction. ResNet includes skip connections and enables a deeper architecture, learning relevant complementary features with better accuracy. DenseNet includes four dense blocks where each layer obtains inputs and passes its feature maps to the preceding layers. Therefore, the last layer concatenates all the features with collective information and is sent to a classification module. These classification model frameworks were altered by replacing the final layer to perform binary classification. For the 2D model training, we segmented the 3D tumor volume, created slices of size 45×45 , and stuck them side by side to make an input image of size 225×225 . These input images are then fed for model training and validation.

## 4. Gated Feature Fusion

In our framework, we incorporated a late fusion technique to effectively merge multi-modal global and local features from individual VOIs for HPV prediction. As demonstrated in Figure 4, the gated fusion operation comprises three layers: feature concatenation, coefficient matrix calculation, and weighted feature fusion. The $F^{CT}\in \;R^{s\times \ell \times w}$ and $F^{PET}\in R^{s\times \ell \times w}$ denote the probability maps of CT and PET features at the fully connected (FC) layer of the ensemble network. The symbol *s* indicates the number of slices, *ℓ* is the height, and *w* is the width of the map. The feature maps $F^{CT}$ and $F^{PET}$ from Figure 4 were concatenated to obtain a fused probability map $F^{fusion}\in \;R^{2s\times \ell \times w}$ . Hereafter, we employed a 3D convolution operation with filter weights $W\in R^{n\times 2s\times 1\times 1}$ , where *n* is the number of filters with each filter dimension of 2s×1×1 . During the training, filter weights were learned to correlate the two feature maps from individual CT/PET image regions and determine their complementary contributions to the final HPV classification. Therefore, the output of the last Conv layer was a coefficient matrix $M\in R^{s\times \ell \times w}$ and described as:

(1) $$\begin{array}{c} M_{k,i,j}=\sum_{t^{\prime }=1}^{2s}F_{t^{\prime },i,j}^{fusion}\times W_{t^{\prime },k,i,j}\\ \forall k\in [1,s],i\in [1,\ell ],j\in [1,w]. \end{array}$$

Finally, a softmax squashing function was applied to matrix *M* and mapped the $M_{k,i,j}$ values in the range ∈[0,1] . We term the $M^{CT}=M$ and $M^{PET}=\left(1-M\right)$ as weighted gates and represent two coefficient matrices. Therefore, $M_{k,i,j}^{CT}$ and $M_{k,i,j}^{PET}$ work as regularizers for the loss function by penalizing the weights during the training step and denote how output can rely on CT and PET feature maps to predict the pixel (i,j) in slice *k*. The two coefficient matrices are then applied to weigh the contribution of each modality as follows:

(2) $$\begin{array}{c} {\tilde{F}}^{CT}=F^{CT}⊙M^{CT}\\ {\tilde{F}}^{PET}=F^{PET}⊙M^{PET} \end{array}$$

where ⊙ denotes the element-wise product dot product (Hadamard product). Finally, we generated a gated fusion probability feature map as a weighted combination of $F^{Cur}$ and $F^{Ref}$ and leveraged it to optimize the loss function via stochastic gradient descent.

(3) $$\begin{array}{c} {\tilde{F}}^{fusion}={\tilde{F}}^{CT}+{\tilde{F}}^{PET} \end{array}$$

## 5. Experiments

For our deep learning classification pipeline, a cohort of 404 subjects was selected from the TCIA online datasets from diverse institutes. The data allocation summary is provided in Figure 1. After pre-processing, the data were fed to train our ensemble architecture, facilitating simultaneous feature extraction from primary tumor lesions and predicting HPV status (HPV-positive or HPV-negative). The input image data are normalized and standardized across modalities. The training set was split by class status at the patient level. The 30 MAASTRO PET and contrast-enhanced CT scans are kept aside and used for external validation. A sample of 80 patients was selected from 404 training subjects and kept aside for independent validation of each ensemble-based model. The remaining 324 subjects were used for model training, hyperparameter optimization, and cross-validation.

**Figure 5:**
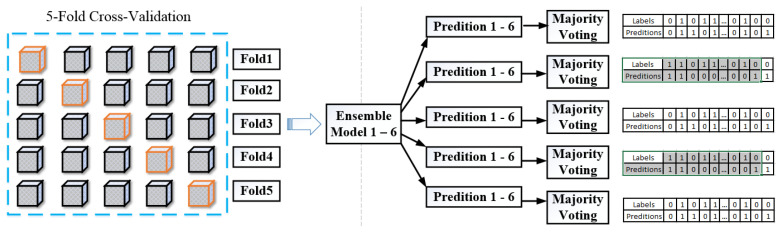
Five-fold cross-validation and a brief outline of our ensemble prediction through major voting. On the extreme right, we compared ground truth results with model predictions.

We included a Scikit-learn-based library for five-fold validation to access the model’s performance and reduce model bias. The model training was performed on sub-sets of the input data (80%) and evaluated on a complementary subset of validation data (20%). In each cross-validation round, the training folds were standardized to avoid information leakage to the validation folder, followed by the model training. The outcome result was evaluated in all the validation folds and averaged over all epochs to produce stable performance (Figure 5). We trained the ensemble model six times for each fold with different weight initialization and aggregated each model’s prediction probability. Subsequently, with six models and five-fold cross-validation, we ended up with thirty predictive scores for each source imaging modality. The class output of each model was averaged for each fold and voted to deduce the final prediction level. For each source imaging modality and VOI combination, the class that retrieved the highest average probability was reported as the final ensemble output. The above feature learning was repeated for each CT and PET image/patch in our dual ensemble network. For our model analysis, we performed both weighted soft voting and majority voting for the final classification. Since we have developed an ensemble architecture comprising six deep learning models (classifiers), each model within the ensemble provides class predictions. In the context of majority voting, the maximum prediction is determined by adding individual predictions that are correctly classified and, after that, taking the majority vote. Soft voting considers the confidence of each classifier’s prediction. Each classifier assigns a probability score to an individual class (binary). Finally, the ensemble’s prediction is evaluated based on the highest average class probability scores across the models. Our model was trained using binary cross-entropy loss, as given below:

(4) $$\begin{array}{c} L_{p}\left(q\right)=-\frac{1}{n}\sum_{k=1}^{n=2}y_{i}\times \log p\left(y_{i}\right)+\left(1-y_{i}\right)\times \log\left(1-p\left(y_{i}\right)\right) \end{array}$$

where *n* is the number of classes with a predicted probability *p* or (1−p) , and *y* is the ground truth label 0 or 1 for our binary classification.

### Training Setup

**Figure 6:**
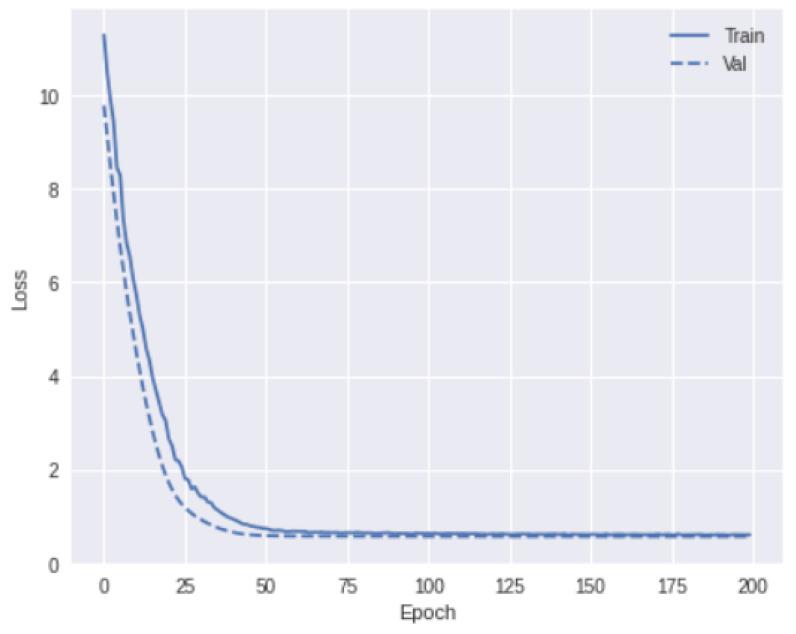
The convergence of loss function over training and validation data over epochs.

Our 3D ensemble model implementation was derived from the Python libraries and Tensorflow-based deep learning framework. Figure 3 and Figure 4 show the schematic diagram of our training pipeline. All the experiments were carried out on a desktop computer and NVIDIA RTX A6000 GPU graphic card with a RAM of 40 GB. The localized segmented primary tumor region in each training subject was decomposed into *N* number of non-overlapping 3D patches and put into a set S = { $S_{i}$ }, i∈(1…N) . A patch size of 16×16×16 was chosen judiciously to accommodate at least 50% overlap of the tumor region and we input those into the model training. To avoid training time over-fitting and increase model generalizability, the input data were augmented with image rotation by an angle of $45^{\circ }$ and $135^{\circ }$ , scaled with a random factor between 0.4 and 0.8, Gaussian noise was added, and image brightness and contrast were changed. The batch size was set to 32. At each epoch, the errors were back-propagated to minimize the loss function, and layer-wise weights were updated in terms of gradients and learning rates. During the training, we used a learning rate ( η ) of $1\times 10^{-5}$ in the last layers and $1\times 10^{-4}$ in the remaining layers for better convergence (Figure 6). The learning rate was divided by 100 when the loss value stabilized. We partitioned the data into five folds and performed repeated cross-validations to assess unbiased model performance. Our training data have a substantial number of imbalances in the target class distribution, with more HPV-positive (75%) samples than HPV-negative ones (25%). To overcome this data skewness, we adopted stratified cross-validation to ensure that the same proportion of labels was effectively retained in each training and validation fold.

## 6. Results and Analysis

The bioimaging patterns like tumor texture, shape, and hyper-metabolism can provide additional information regarding HPV status in OPSCC. HPV-associated OPSCC has distinct biological and clinical characteristics compared to HPV-negative cases. Therefore, identifying the incidence of OPSCC association with HPV infection itself is important. Our model offers noticeable AUC, precision, recall, and specificity gains even using a small-scale ensemble model with just 4.5 k parameters. More importantly, we demonstrate that using a shallow 3D ensemble model can be effective compared to other state-of-the-art models, encouraging practical learned HPV prediction models by resolving its fundamental challenges.

### Evaluation

We report the performance of our model trained on 404 labeled images. The performance of our HPV classification model was evaluated in terms of precision, recall, True Positive Rate (TPR), False Positive Rate (FPR), $F_{1}$ score, and receiver operating characteristic (ROC) curve. We calculated true positive (TP), false positive (FP), false negative (FN), and true negative (TN) values for each patch through different iterations. The TP value was computed as the number of instances correctly identified by our model. To understand our binary classification performance, we calculated the metrics below using various prediction probability threshold cut-offs in the range [0–1].

$$Precision=\frac{TP}{TP+FP},\;Recall=\frac{TP}{TP+FN}$$

$$F_{1}=2\times \frac{Precision\times Recall}{Precision+Recall},\;Specificity=\frac{TN}{TN+FP}$$

**Figure 7:**
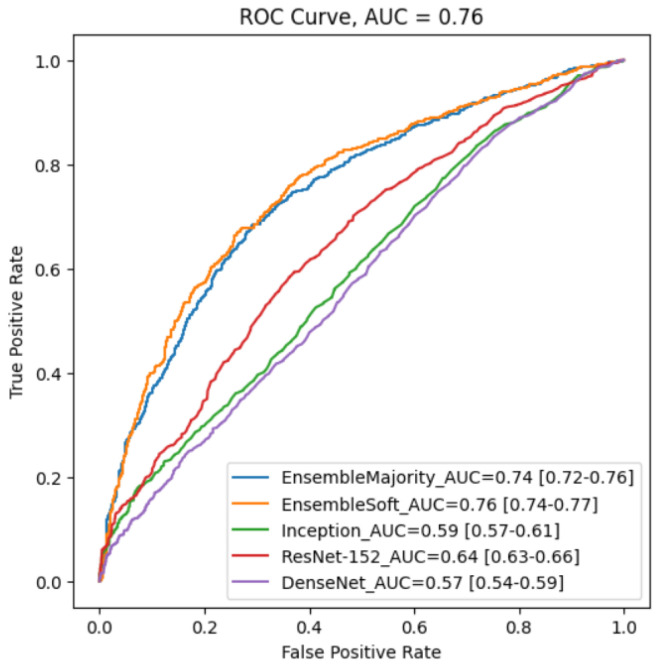
Five ROC curves generated by different classification models using an external validation dataset. Our ensemble model with soft voting performs the best with an AUC of 0.76.

Table 2 and Figure 7 depict the model’s classification performance and ROC curves by TPR versus FPR at various threshold levels. The Youden index analysis summarizes the ROC curve statistic between the true positive rates and false positive rates of our HPV classification. At the ROC curve’s optimal threshold, we determined the classifier’s sensitivity and specificity and calculated the Youden Index (J) as 0.62 using the formula J = Sensitivity + Specificity − 1 [33]. On the other hand, AUC measures the area under the entire ROC curve and provides aggregate performance across all possible classification thresholds [34]. A higher AUC value represents a better prognosis prediction for HPV classification. We also computed the area under the receiver operating characteristics curve (AUC) of our ensemble soft voting and majority voting model as 0.76 and 0.74, respectively (Table 2). Our ensemble models performed significantly better than other traditional 2D models. The potential reason for better output in the soft voting classification setting turns out to be the inclusion of unequal weight hyper-optimization, which balances out the individual base models’ weaknesses in the dataset.

**Figure 8:**
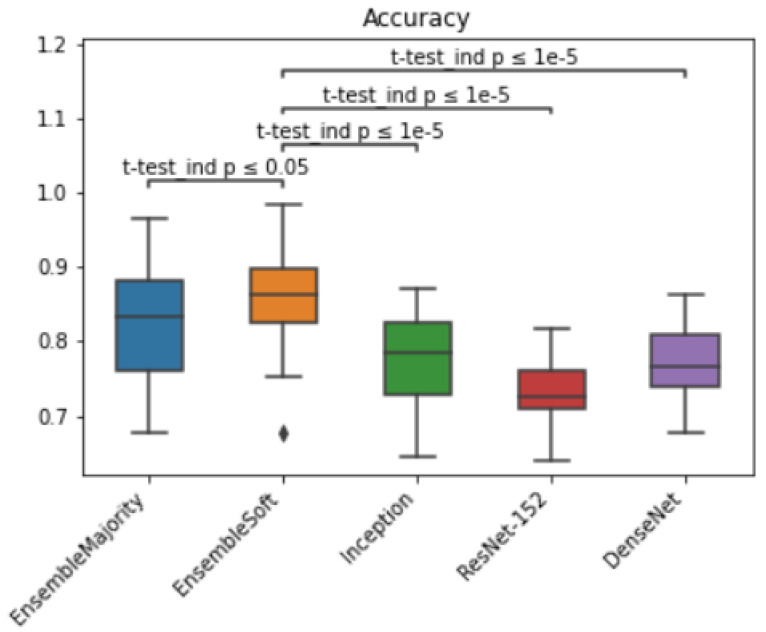
Box plot for HPV classification accuracy results and *t*-test statistical significance comparison across models on eighty independent validation cohorts.

The $F_{1}$ score represents the harmonic mean of precision and recall by taking both metrics into consideration. As the class distribution was highly unbalanced in our dataset, we quantitatively measured the $F_{1}$ score of our method and compared it with other methods (Table 2). Besides that, we compared the statistical significance of our model accuracy by calculating the t-test among models and reported *p*-values < 0.05 (Figure 8). The ensemble model with soft voting performed the best, with a median value of 0.86 and within a 95% confidence interval (CI: 0.83–0.88) when evaluated on an independent dataset.

We reported the mean and standard deviation (SD) of the AUC values of different ensemble CNN predictors across five validation folds and repeated them for CT and PET inputs. To indicate the effectiveness of our ensemble model, we employed the Keras-based pre-trained deep learning models Inception [30], residual network ResNet–152 [31], and DenseNet [32] to CT and PET and compared the results. As shown in Table 2, our model achieved the highest AUC score among all models. Among 2D models, the ResNet model predicted better performance and showed the lowest AUC value in DenseNet. In predicting HPV status, our model achieved 87% (SD 0.0421) mean accuracy, 0.754 (SD 0.062) mean area under the ROC curve, 0.718% (SD 0.0655) mean specificity, and 0.705% (SD 0.0438) mean sensitivity. The results of each fold are shown in (Table 3). In our binary classification, we estimated each class’s weighted $F_{1}$ score and took the average. The high $F_{1}$ score of our model indicates that it has a low misclassification rate. Our proposed model achieves 0.746 $F_{1}$ score values (Table 2) and AUC of 0.76 on the independent validation set. The 71.8% recall value indicates that very few FNs were predicted as HPV negative and implies better prediction sensitivity of our model. Finally, the precision value of our model assures that of the patients who were predicted as HPV-positive, 74% actually had OPSCC associated with HPV.

## 7. Discussion

Human papillomavirus (HPV)-related head and neck cancer cases have increased in recent decades. This work investigated a new ensemble deep learning framework for identifying HPV presence in Oropharyngeal Squamous Cell Carcinoma (OPSCC) and delineating its extent in primary tumors. The ensemble network includes a cascade of two parallel 3D deep learning pipelines for PET and CT volumes. These pipelines independently learn discriminative features from various deep learning classifiers and finally fuse them for understanding OPSCC sub-typing. To reduce model prediction variance, our architecture combined six CNN models with multiple layers of nodes. We used a gated fusion technique that comprises three layers: feature concatenation, coefficient matrix computation, and weighted feature fusion. By using this method, the network learns filter weights during the training process by comparing the two individual feature maps received from PET and CT images. It also determines their complementary characteristics.

To compare our results, we also trained Inception V3, ResNet–50, and DenseNet 2D models. Our models outperformed traditional 2D models significantly, as presented in Results and Analysis (Section 6). Our ensemble multi-modal feature fusion technique achieved higher classification performance than single-modality models with an AUC score up to 0.76, suggesting potential benefits from combining features. Evaluation on an independent dataset demonstrated that the ensemble model with soft voting performed the best with a median value of 0.86 and within a 95% confidence interval (CI: 0.83–0.88). We trained and evaluated our model with multi-institutional cohorts and demonstrated sufficient model accuracy for detecting HPV presence in OPSCC, which can provide preliminary assessment before biopsy. We performed single-modality ensemble model analysis and showed that CT images yielded similar classification results to PET images in model accuracy and AUC values.

Cervical lymph nodes are more prone to metastatic malignant tumors and spread from the primary node to other head and neck regions. In the current implementation, we have not included the volume segmentation on lymph node region for predicting HPV association and left it for future research. We expect that the proposed ensemble model may guide future research into survival analysis for the prognosis of distant metastasis and cancer staging in HNSCC. In the future, we will validate the model with larger cohorts and efficient nnU-Net model segmentation [35] technique for tumor localization and use Spatial Pyramid Pooling fusion techniques [22] to fine-tune the low-label texture features for HPV classification.

Our results strengthen the idea of using deep learning methods to extract intricate patterns and features from multi-modal medical imaging data. In the future, it may offer a level of precision that surpasses traditional manual methods. Deep neural networks’ ability to automatically learn and adapt from imaging datasets can enable them to discern subtle abnormalities indicative of cancer and other diseases, even in the early stages. This fusion of multi-modal medical imaging and advanced deep learning architecture holds immense promise for improving the sensitivity and specificity of cancer detection.

## 8. Conclusions

In this work, we have proposed an effective 3D deep learning ensemble framework that combines features from PET and CT images for OPSCC malignancy prediction and HPV classification. HPV is one of the primary risk factors for OPSCC. Manually segmenting small tumor regions on medical images and then classifying them into different HPV sub-types is challenging in the field of oropharyngeal cancer. In this work, the HPV detection problem was framed as a machine learning design problem for characterizing local features and understanding disease severity. PET and CT images and metadata of 404 patients from diverse institutes were collected as part of our OPSCC data collection process. We developed an image prepossessing pipeline and created tumor volume segmentation masks. We separately segmented CT and PET volumes of interest (VOIs) in the primary tumor regions. The segmented volume had a size of 45×45×45 .

Our ensemble architecture comprises five convolutional layers, ReLU, Batch Normalization, and linear combinations of max and average pooling, drop out, and fully connected modules before feature concatenation in softmax classification. An ensemble of 3D CNN models was used for learning multi-modal features from individual PET and CT images in order to identify intra-tumor heterogeneity. The fused features were finally used for disease classification. Multiple CNN models were created using different dropout and learning rate values for layer-wise node variation. A feature concatenation layer assembled the discriminative patch representations and fused them to another intermediate vector. Then the model performed binary classification with a soft-max classifier. To extract dense feature maps from multiple sequences, we used a weighted gate fusion technique. Multi-instance learning techniques are used in our training, and multiple patches are created from tumor regions. Each patient’s data were therefore treated as a bag of patches with each patch representing an instance of a tumor zone.

We sampled 80 patients from the 404 patients and kept them aside for independent validation of the ensemble-based models. With the remaining 324 patients, we trained the model, optimized the hyperparameters, and performed cross-validation. As part of our model training procedure, we cross-validated five folds with stratification, evaluated sampling variability with standardization, and pre-processed data to minimize ensemble model bias. We trained our ensemble architecture on the pre-processed data, facilitating simultaneous feature extraction from primary tumor lesions and HPV status prediction (positive or negative).

HPV classification performance of our model was evaluated in terms of precision, recall, True Positive Rate (TPR), False Positive Rate (FPR), $F_{1}$ score, and receiver operating characteristic (ROC) curve. Based on the results from experiments, the multi-modal ensemble model with soft voting outperformed the single-modality (PET or CT) model. Our method achieved 0.746 $F_{1}$ score values and AUC of 0.76 on the independent validation dataset. Furthermore, the 71.8% recall value indicates that only a small percentage of FNs were predicted as HPV-negative, which suggests better prediction sensitivity. The precision value of our model confirmed that 74% of the HPV-positive patients actually had OPSCC related to HPV.

The developed ensemble feature fusion architecture using multi-modal CT and PET images provides superior results in differentiating the HPV association in OPSCC compared to uni-modal deep learning models. Due to the small dataset size, model performance was not high enough to replace a biopsy. However, training the model with a larger dataset and a more diverse population might further improve performance. 

## Figures and Tables

**Table 1:** Patient information and imaging characteristics of training, independent validation, and external validation cohort.

Patient Information		Training Cohort	Independent Validation (Cohort)	External Validation (Cohort)	*p*-Value Training vs. Independent	*p*-Value Training vs. External
Number of Patients		324	80	30		
Sex (%)					0.76	1
	Male	270 (83.33%)	65 (81.3%)	24 (82.8%)	N/A	
	Female	54 (16.67%)	15 (18.8%)	5 (17.2%)		
Age in Years (mean, SD)		61.54 (9.28)	61.47 (8.14)	62.06 (5.81)	0.77	0.81
HPV Status (%)	Positive	242 (74.69%)	60 (75.0%)	12 (40%)	N/A	
	Negative	82 (25.3%)	20 (25.0%)	18 (60%)		
PET (mean, SD)	Slice thickness (in mm)	3.38 (0.36)	3.32 (0.33)	3	N/A	
	In-plane pixel spacing (in mm)	4.34 (0.91)	4.36 (0.92)	3		
	In-plane image matrix (N × N)	150.52 (60.44)	150.74 (61.55)	256 × 256		
CT (mean, SD)	Slice thickness (in mm)	3.14 (0.55)	3.28 (0.41)	N/A	N/A	
	In-plane pixel spacing (in mm)	1.12 (0.18)	1.13 (0.18)	N/A		
	In-plane image matrix (N × N)	512 × 512	512 × 512	N/A		

**Table 2:** Performance comparison of HPV classification over primary tumor VOI sources and result comparison over other state-of-the-art models on a test dataset of 80 subjects (independent validation).

Imaging Modality	Model (Source Region)	Voting	Accuracy	Precision	Recall	$F_{1}$ Score	AUC (SD)
CT + PET	3D Ensemble + VOI	Majority	0.84	0.756	0.705	0.729	0.74 (0.064)
CT + PET	3D Ensemble + VOI	Soft	0.86	0.804	0.717	0.746	0.76 (0.055)
CT-only	3D Ensemble + VOI	Soft	0.81	0.723	0.682	0.702	0.672 (0.068)
PET-only	3D Ensemble + VOI	Soft	0.76	0.693	0.705	0.698	0.658 (0.065)
CT + PET	2D Inception [30] + ROI	Majority	0.74	0.673	0.635	0.653	0.641 (0.082)
CT + PET	2D ResNet [32] + ROI	Majority	0.78	0.682	0.694	0.687	0.652 (0.091)
CT + PET	2D DenseNet [31] + ROI	Majority	0.71	0.614	0.635	0.624	0.627 (0.078)

**Table 3:** Prediction outcome of an ensemble of six base models among different folds and metrics and comparison. The model performance was collected from an external validation dataset of thirty patients. Average values are reported across folds.

Model	Metrics	Fold 1 (%)	Fold 2 (%)	Fold 3 (%)	Fold 4 (%)	Fold 5 (%)	Average
Ensemble + CT + Majority Voting	Accuracy	84.42	81.45	82.54	83.51	80.58	82.51
Ensemble + PET + Majority Voting	Accuracy	80.67	79.93	80.28	83.27	82.05	81.21
Ensemble (CT + PET) + Majority Voting	Accuracy	88.53	86.14	82.17	84.25	81.18	84.45
Ensemble Model + (CT + PET) + Soft Voting	Accuracy	86.55	89.93	85.14	83.01	86.25	86.17
Inception Model	Accuracy	78.34	75.15	75.11	76.23	70.51	75.06
ResNet Model	Accuracy	75.06	73.89	75.11	476.23	71.85	73.39
DenseNet Model	Accuracy	81.24	77.48	79.64	80.53	81.48	80.07
Ensemble Model + Soft Voting	Area under ROC	0.678	0.702	0.654	0.675	0.709	0.754
Ensemble Model	Specificity (1 − FPR)	0.705	0.694	0.741	0.725	0.728	0.718
Ensemble Model	Sensitivity (TPR)	0.715	0.698	0.676	0.688	0.737	0.705

## Data Availability

Publicly available datasets were analyzed in this study. This data can be found here: [23,24,25,26].
